# Subacute Stent Thrombosis in a Patient With COVID-19 Despite Adherence to Antiplatelets

**DOI:** 10.7759/cureus.13194

**Published:** 2021-02-07

**Authors:** Karim O Elkholy, Asma Khizar, Abdullah Khan, Narek Hakobyan, Sonu Sahni

**Affiliations:** 1 Internal Medicine, Brookdale University Hospital Medical Center, Brooklyn, USA; 2 Cardiology, Brookdale University Hospital Medical Center, Brooklyn, USA; 3 Internal Medicine, Saba University School of Medicine, Brooklyn, USA; 4 Research Medicine, New York Institute of Technology College of Osteopathic Medicine, Old Westbury, USA; 5 Primary Care, Touro College of Osteopathic Medicine, New York, USA

**Keywords:** cardiac catheterization, st-elevation myocardial infarction (stemi), corona virus, covid 19, stent thrombosis, antiplatelet therapy, coronary artery disease, pci, primary pci, cardiac troponin

## Abstract

The coronavirus disease 2019 (COVID-19) creates a significant burden on the cardiovascular system. Moreover, diagnosing coronary artery disease in patients with COVID-19 may be clinically challenging. Herein, we present a case of in-hospital stent thrombosis and thrombotic occlusion of the right coronary artery after initial revascularization and adherence to antiplatelet therapy.

## Introduction

The novel coronavirus disease 2019 (COVID-19) infection has been associated with complications secondary to coronary artery disease (CAD), with ST-elevation myocardial infarction (STEMI) being the most serious as it is associated with a significantly higher rate of in-hospital mortality [1]. The COVID-19 pandemic posed a serious threat to public health beyond its direct characteristics of virulence and infectivity. It was presumed that patients enduring chest pain would not present to emergency rooms due to the ongoing pandemic, which is evident by reports confirming unusually lower rates of hospitalizations due to myocardial infarctions during the height of the pandemic [2]. This is an unfortunate case of a 48-year-old male enduring acute STEMI, which was treated with thrombolysis followed by rescue percutaneous coronary intervention (PCI). In this case, we highlight the importance of recognizing the risks of early stent thrombosis in patients with COVID-19 presenting with myocardial injury in addition to identifying the serious thrombogenicity of COVID-19 and understanding the outcomes of stent thrombosis in patients with COVID-19.

## Case presentation

**Figure 1:**
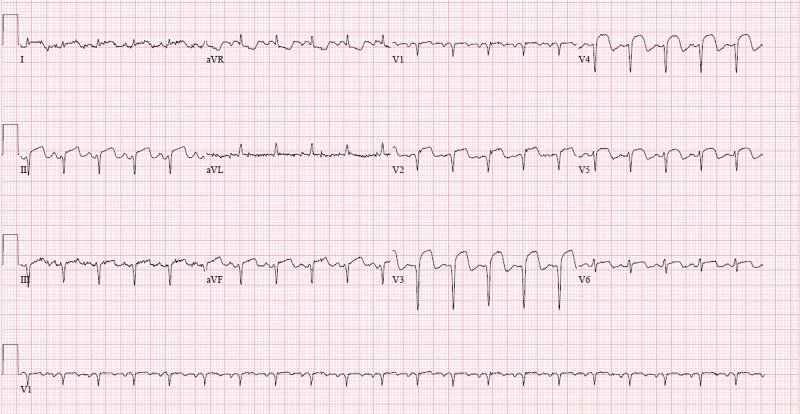
Initial insult STEMI. EKG showing ST-segment elevation in leads II, III, and V2-V5. STEMI: ST-elevation myocardial infarction; EKG, electrocardiography

**Figure 2:**
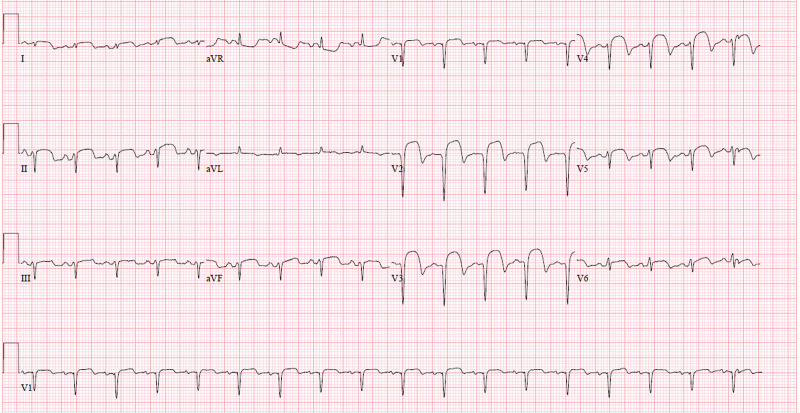
STEMI. EKG showing persistent ST-segment elevation in leads II, III, and V2-V5. STEMI, ST-elevation myocardial infarction; EKG, electrocardiography

**Figure 3:**
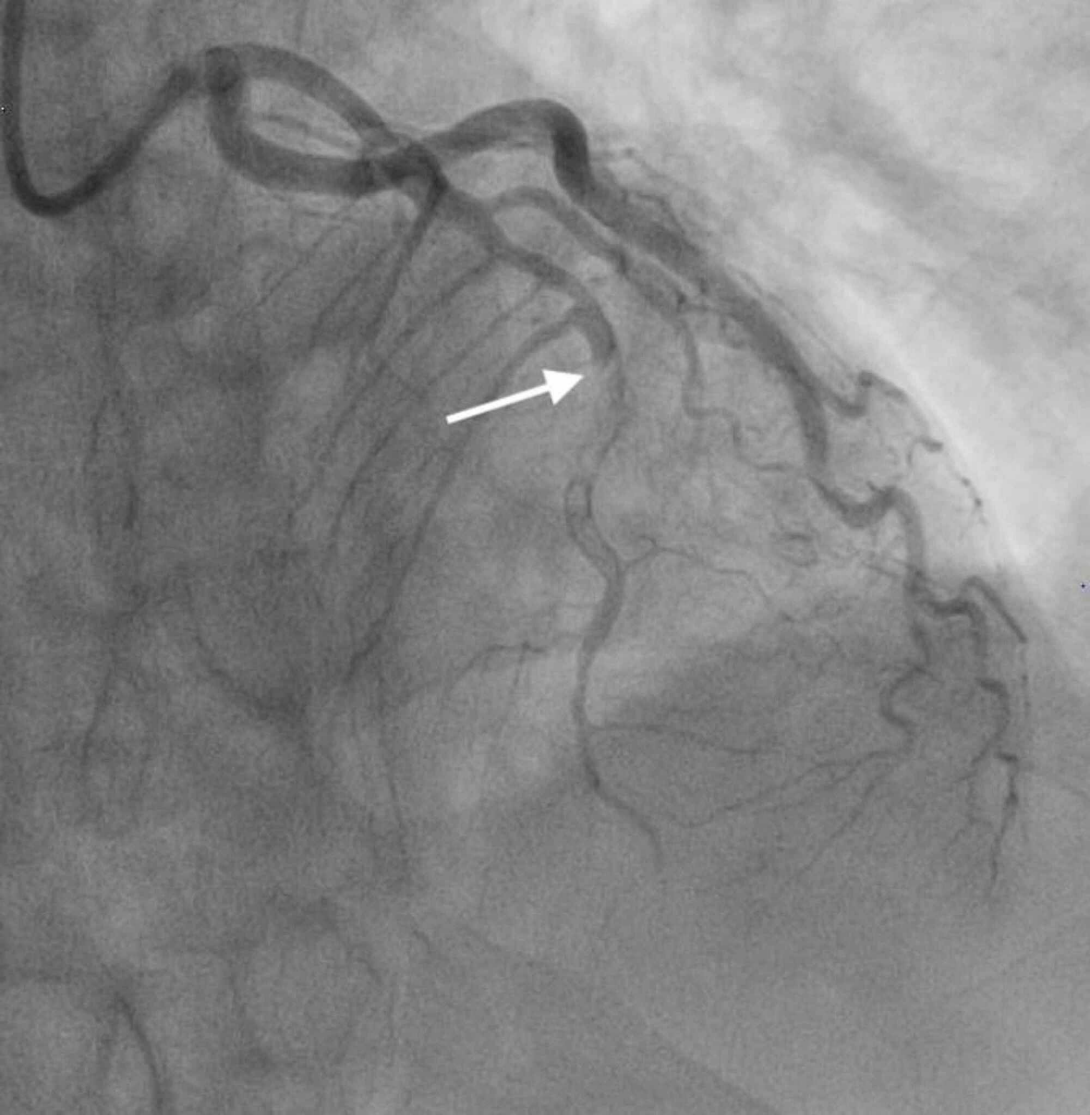
LAD thrombosis. Angiogram image showing thrombosis in the mid-LAD (notice abrupt discontinuation of the contrast material; white arrow). LAD, left anterior descending artery

**Figure 4:**
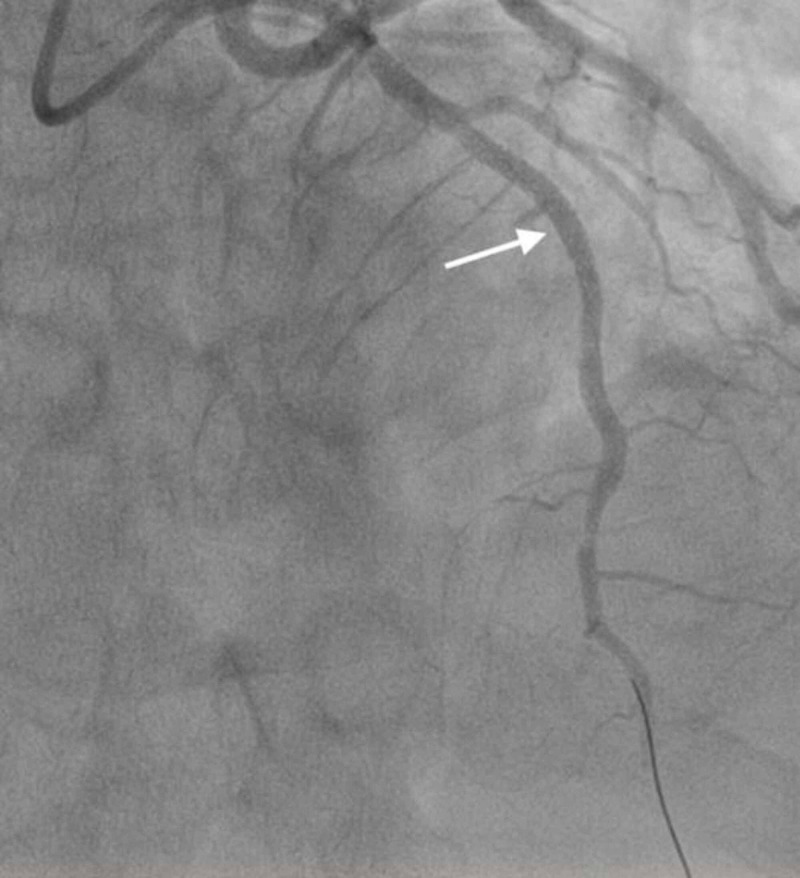
LAD following intervention. Angiogram image showing the LAD following angioplasty and stent placement (notice restoration of the contrast material; white arrow). LAD, left anterior descending artery

**Figure 5:**
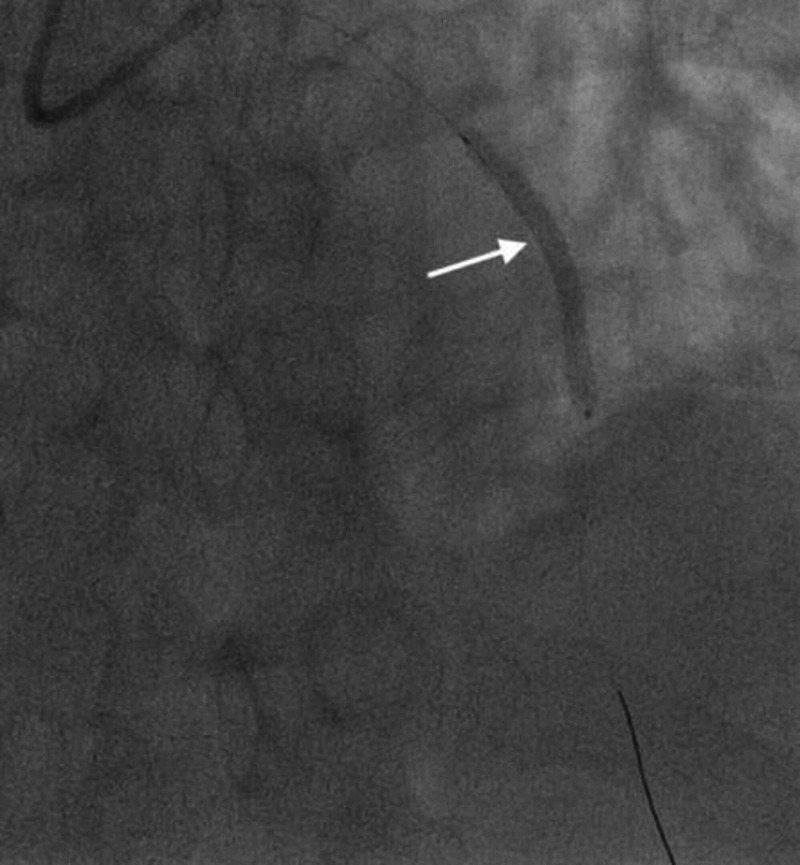
Second-generation DES. Angiogram image showing the DES placed in the mid-LAD (notice shadow of the stent placed; white arrow). DES, drug-eluting stent; LAD, left anterior descending artery

**Figure 6:**
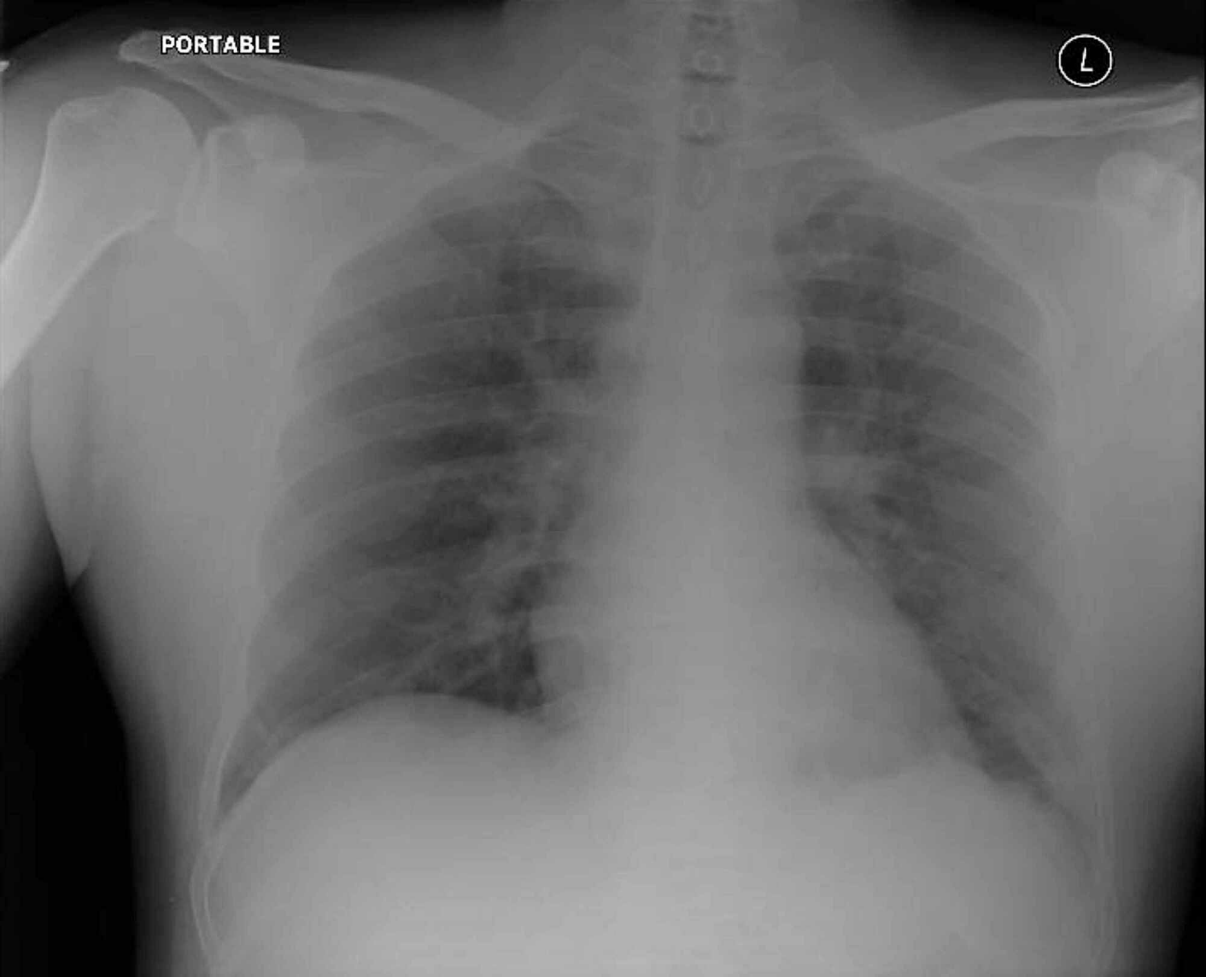
Chest X-ray following COVID-19 diagnosis. Chest plain radiography showing mild bilateral interstitial infiltrates.

**Figure 7:**
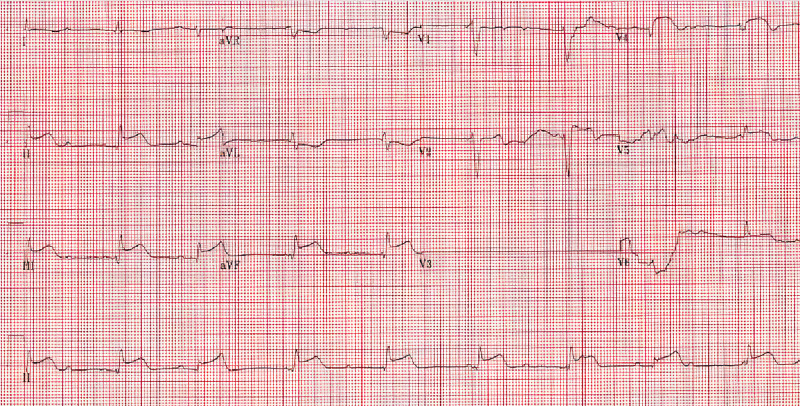
Second insult STEMI. EKG showing complete heart block along with ST-segment elevation in leads II, III, aVL, aVF, V1, and V2. EKG, electrocardiography

**Figure 8:**
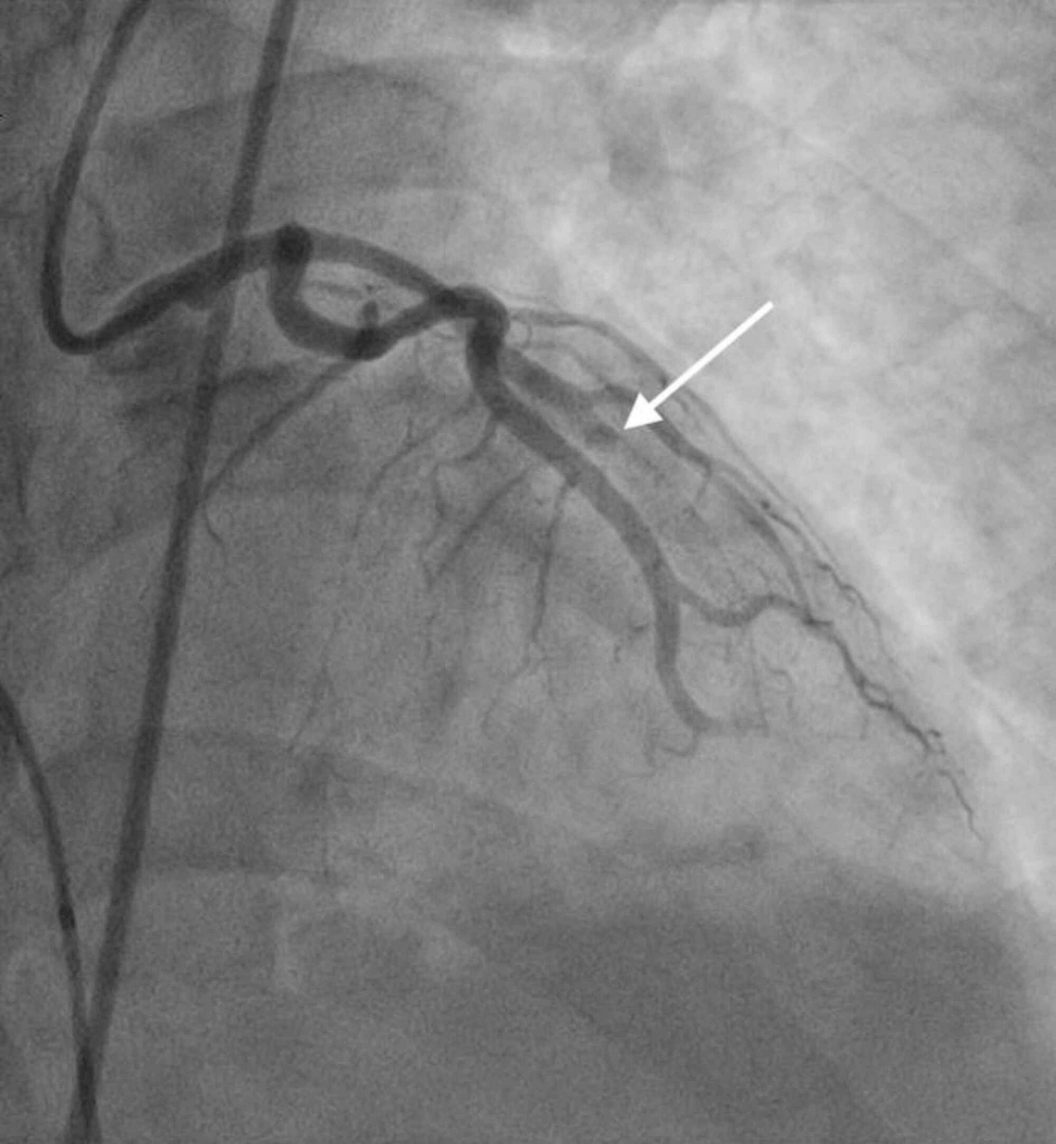
LAD stent thrombosis. Angiogram image shows thrombosis in the LAD (notice abrupt discontinuation of the contrast material; white arrow). LAD, left anterior descending artery

**Figure 9:**
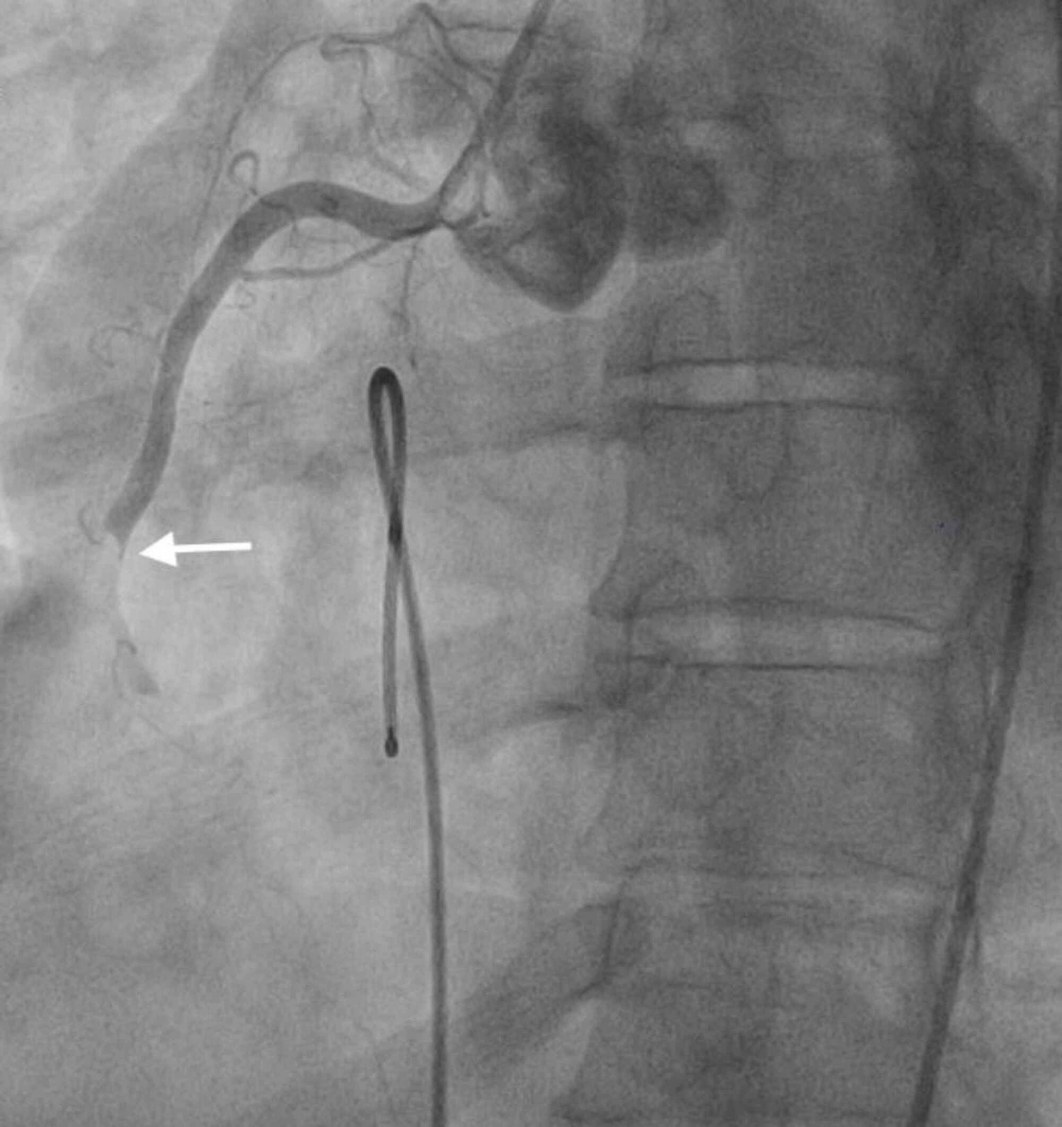
RCA thrombosis. Angiogram image shows thrombosis of the mid-RCA (notice abrupt discontinuation of the contrast material; white arrow). RCA, right coronary artery

**Figure 10:**
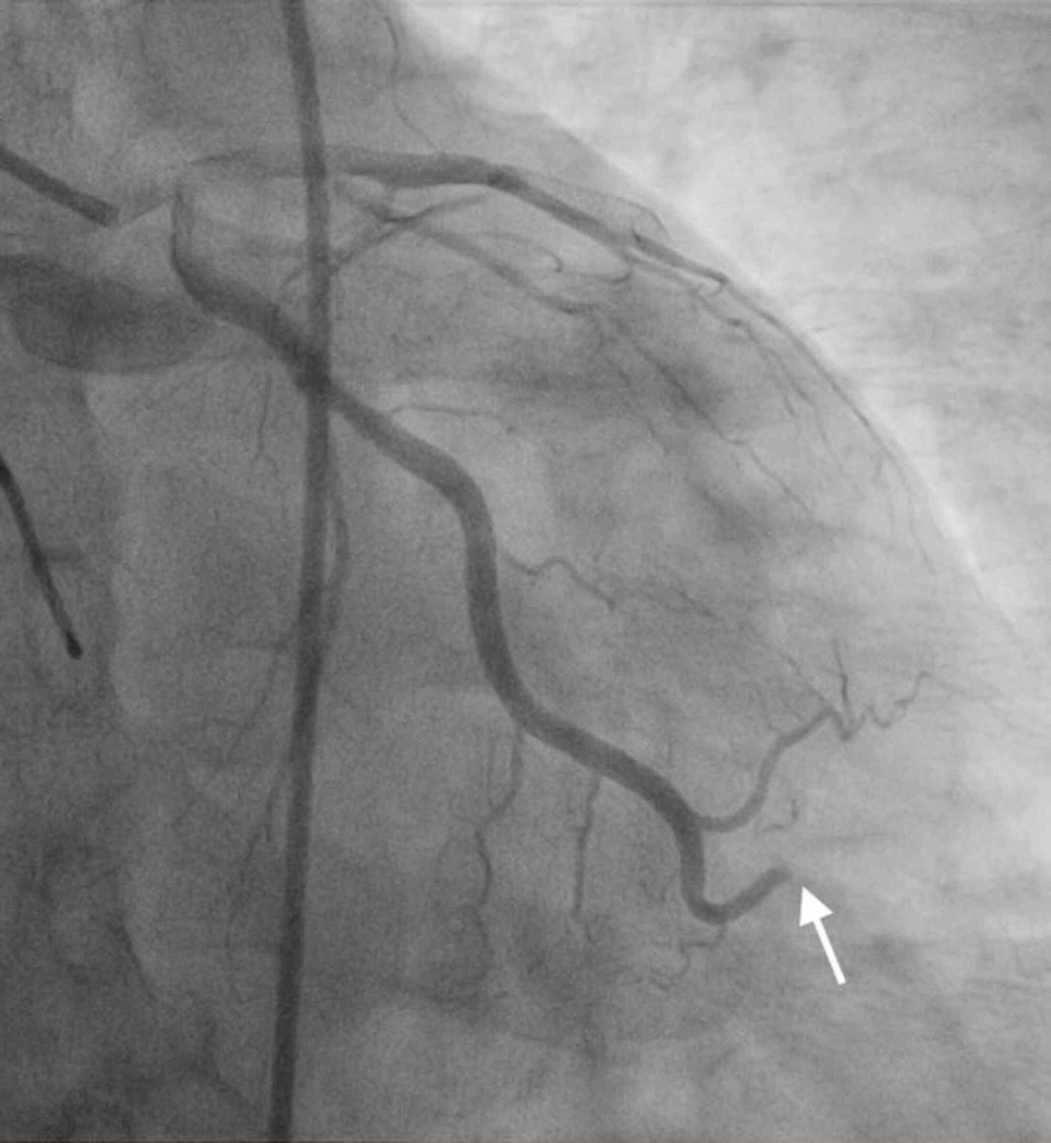
OM1 thrombosis. Angiogram image shows disease in the distal OM1 (notice abrupt discontinuation of the contrast material; white arrow). OM1, obtuse marginal artery 1

**Figure 11:**
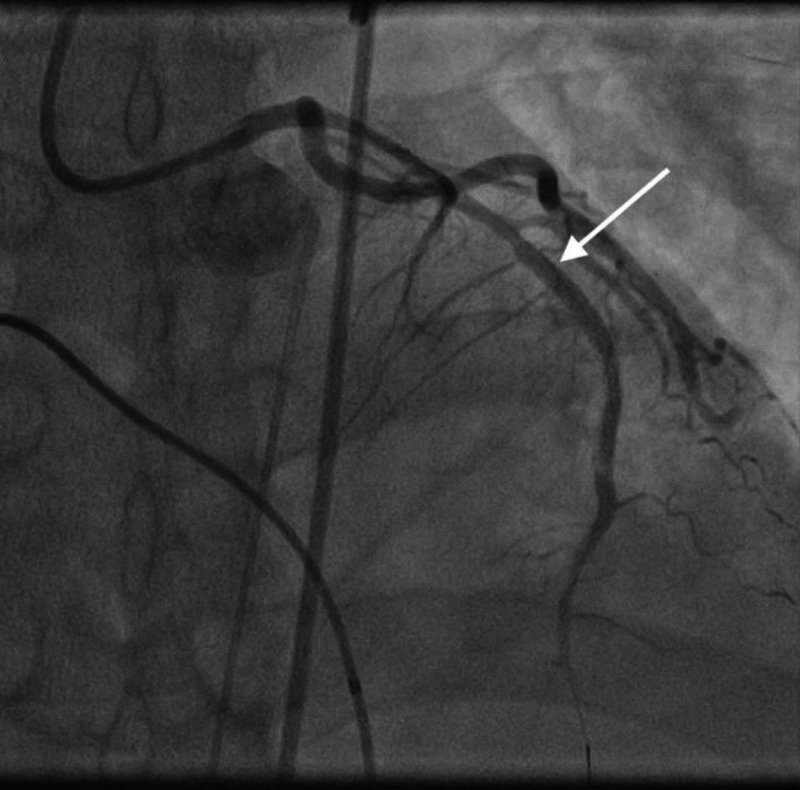
LAD stent thrombosis following angioplasty. Angiogram image of the LAD following angioplasty (notice restoration of the contrast material flow; white arrow). LAD, left anterior descending artery

**Figure 12:**
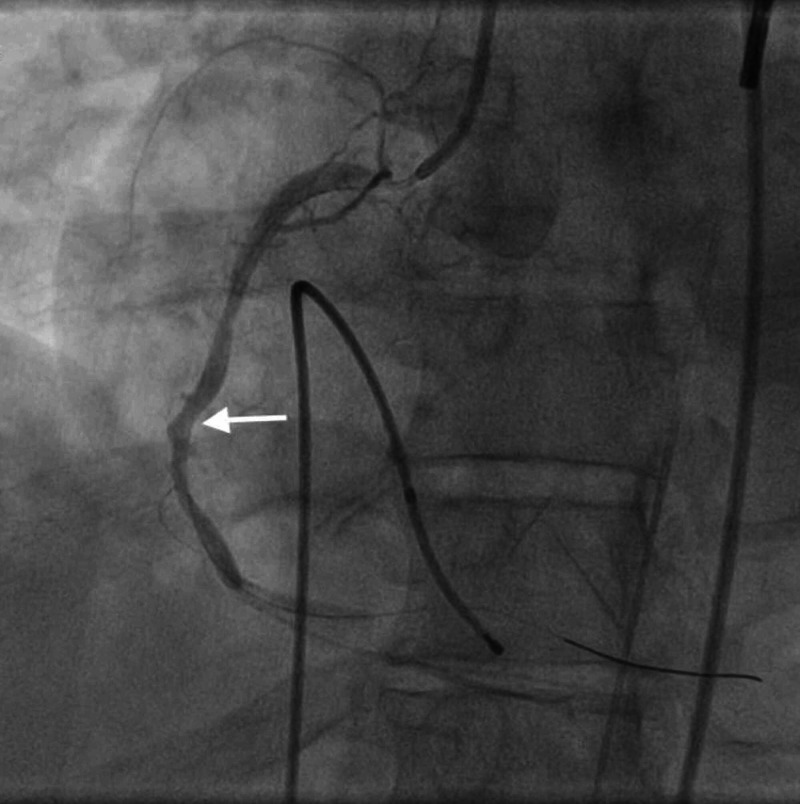
RCA following thrombectomy. Angiogram image shows the RCA following thrombectomy (notice restoration of the contrast material flow; white arrow). RCA, right coronary artery

We present a case of a 48-year-old male who initially presented to the hospital with a chief complaint of pleuritic chest pain accompanied by fevers, dry cough, and headache for several days. The patient had a medical history of insulin-dependent diabetes mellitus, hypertension, and mixed hyperlipidemia. He is also a former smoker. On arrival, his vital signs revealed a blood pressure of 118/62 mmHg, pulse of 95 beats/minute, breathing rate of 20 breaths/minute, and oxygen saturation of 96% while breathing ambient air. Initial electrocardiography (EKG) showed ST-segment elevations in leads II, III, and V2-V5 (Figure 1). Along with hyperglycemia, laboratory workup revealed an elevated troponin I (Table 1). The facility which the patient presented to was not a PCI capable facility; therefore, thrombolysis with tissue plasminogen activator (tPA) was attempted, which failed. He was transferred to our facility, which is PCI capable. Repeated EKG at our facility showed persistent ST elevations in leads II, III, and V2-V5 (Figure 2), and laboratory workup showed resolution of his hyperglycemia and elevated troponin I (Table 1). Urgent coronary angiogram was performed, which revealed single-vessel disease in the left anterior descending (LAD) artery due to a large thrombus (Figure 3). Thrombectomy along with placement of a drug-eluting stent (DES) was performed. The successful intervention was confirmed by the restoration of blood flow with thrombolysis in myocardial infarction flow of grade 3 following the intervention (Figure 4), and the remainder of the angiogram revealed normal coronaries. The stent placed was a second-generation zotarolimus-eluting stent measuring 2.75 x 34 mm (Figure 5). Presenting symptoms of fevers and dry cough delineated high suspicion for COVID-19. Nasopharyngeal swab for severe acute respiratory syndrome coronavirus 2 (SARS-CoV2) exhibited positive reactivity, and, as a result, a diagnosis of COVID-19 was confirmed. Chest radiography was obtained that showed mild bilateral interstitial infiltrates (Figure 6). The patient was started on aspirin 81 mg daily, clopidogrel 75 mg daily, atorvastatin 80 mg daily, and metoprolol 50 mg daily. Transthoracic echocardiography (TTE) revealed an ejection fraction of 40-50% with akinesis of the mid-apical, mid-anteroseptal, and apical walls (Video 1). The patient remained in the hospital for the management of SARS-Cov2 infection with hydroxychloroquine 400 mg twice daily on day 1 and then 200 mg daily, in addition to daily vitamin C and zinc supplementation. On day 3 of the hospital stay, the patient endorsed a substernal chest pain; EKG showed complete heart block and ST elevation in II, III, aVL, aVF, and V2-V5 (Figure 7). Considering the patient's blood pressure of 64/32 mmHg, chest pain, and ST elevation on the EKG, he was rushed for an emergent coronary angiogram. It was revealed that the patient had a 100% occlusion of the prior stent placement site (Figure 8), alongside novel 100% occlusion in the right coronary artery (RCA) (Figure 9) and the distal obtuse marginal (OM1) (Figure 10). Bedside TTE was performed that showed severely reduced ejection fraction as well as diffuse hypokinesis (Video 2). Because of the novelty of the coronary artery lesions alongside the stent thrombosis, it was reasonable to exclude local stent placement issues as the etiology for stent thrombosis. Moreover, the patient was adherent to the antiplatelet therapy, which excluded non-adherence as the reason for stent thrombosis. Management of this patient needed a multidisciplinary team of cardiologists, electrophysiologists, cardiothoracic surgeons, infectious disease specialist, pulmonologist, and extracorporeal membrane oxygenation (ECMO) specialist. It was deemed that the patient was not a candidate for ECMO intervention. Given the complete heart block, a transvenous pacemaker was placed. Successful angioplasty of the stent thrombosis was performed (Figure 11), along with thrombectomy of the lesion in the mid-RCA (Figure 12). An attempted intervention was made on the lesion in the distal OM1; however, it was unsuccessful. An Impella® device (ABIOMED, Danvers, Massachusetts, USA) was used and intra-aortic balloon pump was performed to aid in the hemodynamic instability and the presumed cardiogenic shock. The patient endured ventricular fibrillation that was refractory to cardioversion and amiodarone. He later expired.

**Table 1:** Relevant laboratory tests performed. Hemoglobin, Hgb; CRP, C-reactive protein; ESR, erythrocyte sedimentation rate PTT, partial thromboplastin time; INR, international normalized ratio

Laboratory tests	Initial facility	Day 1	Day 3	Reference values
Blood glucose	534 mg/dL	234 mg/dL	194 mg/dL	70–99 mg/dL
Hgb	14 g/dL	12.1 g/dL	9.3 g/dL	12.9–16.7 g/dL
Platelet count	425 x 10^3^/uL	489 x 10^3^/uL	499 x 10^3^/uL	153–328 x 10^3^/uL
Creatinine	-	0.7 mg/dL	1.07 mg/dL	0.66–1.25 mg/dL
Troponin I	0.4 ng/dL	46.4 ng/dL	9.6 ng/dL	0.012–0.034 ng/dL
D-dimer	-	790 ng/mL DDU	-	0–230 ng/mL DDU
CRP	-	6.6	-	0.5–1 mg/dL
Ferritin	-	646 ng/mL	-	17.9–464 ng/mL
ESR	-	72	-	0–15 mm
PTT	-	32.1 seconds	13.5 seconds	23.5–32.5 seconds
INR	-	1.28	1.14	0.7–1.2
Activated clotting time	-	139 seconds	358 seconds	102–142 seconds

**Video 1:** Initial echocardiography showing akinesia of lateral, mid-septal, and apical walls.

**Video 2:** Echocardiography following second STEMI showing severe akinesia of right ventricular wall, septum, left ventricular wall, and apex. STEMI, ST-elevation myocardial infarction

## Discussion

Diagnosing STEMI in patients with COVID-19 has proved challenging, and reports have shown that in patients with COVID-19 presenting with STEMI, a culprit lesion is not always present [3]. Besides, ST-segment elevations in patients with COVID-19 have been attributed to pathologies not related to myocardial infarctions [4]. The exact mechanism of myocardial injury in patients with COVID-19 is not yet completely understood; nevertheless, it is reasonable to think that it could be related to reduced oxygen supply from severe lung injury [5]. Also, the cytokine storm might play a role in the pathogenesis of myocardial injury in COVID-19 patients [6]. Animal models have described the effect of angiotensin-converting enzyme 2 (ACE-2) receptor downregulation on myocardial injury associated with COVID-19 [7].

Intriguingly, stent thrombosis has been associated more frequently with first-generation stents [7]. Acute stent thrombosis as well as late stent thrombosis in patients with COVID-19 have also been reported [8]. However, to the best of our knowledge, this is the first case describing subacute second-generation stent thrombosis in a patient with COVID-19 (Table 2). The mechanism of stent thrombosis is thought to be associated with the thrombogenicity of COVID-19 evident by higher rates of venous thromboembolism, elevated D-dimers, fibrinogen, and low antithrombin levels [9]. Moreover, drug interactions between anticoagulants and investigational therapies for COVID-19 might play an important role [10]. There are no established guidelines for the treatment of patients with COVID-19 who present with STEMI. The decision to proceed with either initial thrombolysis or transfer to a PCI center is multifactorial and will differ in different regions [11]. However, the mainstay of management is similar to treating STEMI in non-COVID-19 situations. Moreover, management decisions should include a multidisciplinary team that merits the presence of cardiologists, pulmonologists, as well as infectious disease specialists [11].

**Table 2:** Classification of stent thrombosis. [12]

Type of stent thrombosis	Early		Late	Very late
	Acute	Subacute		
Timing	Within 24 hours	24 hours to 1 month	1 month to 12 months	More than 12 months

## Conclusions

COVID-19 associated coronary artery thrombotic events carry a significant burden on diagnosing and managing such a condition. STEMI can be challenging to diagnose in patients with COVID-19, as neither EKG changes nor elevated troponin is consistent with obstructive CAD. A multidisciplinary approach should be initiated when dealing with a myocardial injury in patients with COVID-19. To the best of our knowledge, PCI remains the recommended modality when dealing with STEMI in patients with COVID-19. However, recurrent thrombotic events following revascularization merit further assessment of this modality when dealing with this patient population.
